# The Warramiri website: applying an alternative Yolŋu epistemology to digital development

**DOI:** 10.1186/s41039-017-0052-x

**Published:** 2017-07-26

**Authors:** Ben van Gelderen, Kathy Guthadjaka

**Affiliations:** 1 School of Education, Charles Darwin University, Casuarina Campus, Ellengowan Drive, Darwin, Northern Territory 0909 Australia; 2 Northern Institute, Charles Darwin University, Casuarina Campus, Ellengowan Drive, Darwin, Northern Territory 0909 Australia

**Keywords:** Warramiri website, Yolŋu epistemology, Indigenous digital education

## Abstract

The intergenerational transmission of traditional language and culture is at the core of Yolŋu Indigenous knowledge practices. The homeland of Gäwa in remote Arnhem Land, Northern Territory, was established by Warramiri clan kinship networks to provide an appropriate place for this crucial role to continue. Technologies have long played a part in this transmission process, but can databases, websites and other digital storage mediums harmonise with existing Yolŋu epistemological and ontological frameworks? In considering an alternative approach to digital development, we rely on the Yolŋu elements of performative epistemology, multiple perspectives and a fundamental, narrative base. We then apply this approach to the construction of the ‘Warramiri website’ (2011–2015) which houses and structures various resources, outlining its applicability to the current educational practices at Gäwa.

## Introduction

**Fig. 1:**
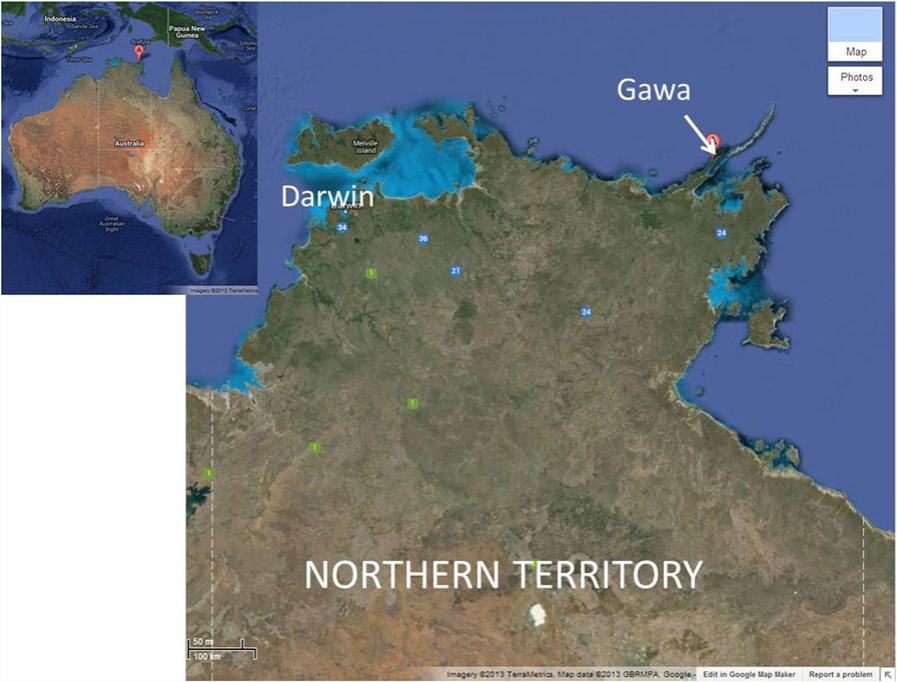
Gäwa, North-east Arnhem Land, Northern Territory

From 2011 to 2015, Kathy Guthadjaka and Ben van Gelderen worked collaboratively on creating the ‘Warramiri Website’. Warramiri is the name of one of the ‘clans’ and language groups of the Yolŋu Indigenous people of North-east Arnhem Land, Northern Territory. Although spread out across this area in various townships, around 40–50 Warramiri members now live at Gäwa, a small, very remote homeland at the tip of Elcho Island. Gäwa was established by Kathy Guthadjaka and her kinship network in the 1980s, literally returning to country by cutting a road through the bush, establishing water supplies and houses, running school classes and eventually, permanently settling there. It is a remarkable story of vision, resilience and Indigenous self-determination (Gäwa Christian School 2016). Specifically, the purpose of establishing Gäwa was to allow young Warramiri to grow up on their ancestral estates; to live ‘on country’ and learn from the land, away from the negative influences and distractions of life in the local township, Galiwin’ku. In short, Gäwa exists to best serve the age-old process of intergenerational transmission of Warramiri language and culture, understood as a holistic, Indigenous foundation (Fig. 1).

The Warramiri have a long history of engaging with outside influences, such as Macassan traders, Christian missionaries and Government authorities, with a sophisticated philosophy of incorporating various elements of new ideas and technologies into their existing ontologies (McIntosh 1994, 1997, 1999; Rudder 1993). Digital technologies certainly fit within this paradigm, and from the school’s outset, Kathy Guthadjaka as a qualified teacher and Elder, employed new technological initiatives to engage and teach young Warramiri of their heritage and inheritance (Charles Darwin University 2006). As part of this drive for the ‘best of both worlds’, in 2004 the community partnered with the Northern Territory Christian Schools Association to provide a permanent, well-resourced school at Gäwa. In 2009, Ben van Gelderen arrived as the first ‘senior’ class teacher and began working with Kathy Guthadjaka in developing and implementing a bilingual and intercultural (‘Bothways’) curriculum for the class. Bothways had been progressively developed at various Yolŋu community schools (Lanhupuy 1988; Wunuŋmurra 1989; Yunupiŋu 1989; Marika-Munuŋgiritj 1990) and highlights the negotiation of Indigenous language and culture priorities within ‘mainstream’ schooling practices as an integrated curriculum. Kathy Guthadjaka had already been working for decades with the Bothways philosophy of education and Ben van Gelderen was keen to incorporate such an appropriate approach that both met the original aims of the Gäwa community and enabled Warramiri students to confidently transition to English usage and experience the breadth of the new Australian Curriculum. But it was in the area of digital resources that a clear potential emerged. Over the years, Kathy Guthadjaka had researched and developed a varied range of resources to facilitate a Warramiri Bothways curriculum. These included electronic story-books, factual audio-recordings on seasons and bush food, women’s crying songs, video songline stories, artworks and modern ‘parables’. There were also sources on Warramiri life in general, published in conjunction with other interested parties, such as music DVDs with “The Wiggles” (Field 2011) and local texts written and filmed from class activities with visiting researchers. These resources were genuinely unique, often stunning pieces, but there was a real concern that time and the elements might conspire to destroy them in such a remote and unrelenting environment. After much discussion, it was agreed that preserving and somehow structuring them into a website could benefit the school and community for generations to come. Such was our initial plan, which seemed so straight forward at the time. As of 2010, we would have answered the fundamental question: ‘Can digitized information feed into, complement, and extend the already well-developed ways that information is handled and managed in Aboriginal communities to support Aboriginal people in doing their knowledge?’ (Verran and Christie 2014, p. 72) with a resounding ‘Yes!’; we were implementing such a process. However, as it turned out, the shift from an array of various digital resources to a structured website was neither practically easy nor theoretically clear, and the more we developed and researched, the more complicated it became.

The transmission of traditional language and culture is both a profoundly religious/historical process and a ‘futures thinking’ activity, to accommodate the interests and technologies of the present and future generations. The great challenge in digital design is to ensure a disjuncture between these two powerful demands does not eventuate; to create respectful and flexible pathways for communities and educators. Our intention in this paper is to outline the key theory postulated by Christie, Verran and other Yolŋu researchers in regards to Yolŋu interaction with digital technologies (of performative epistemology, multiple perspectives and a fundamental, narrative base) and then briefly share the creation of the Warramiri website as a problematised and ongoing application of this theoretical framework.

## Methods

The Warramiri website as a digital resource to aid transmission of Indigenous language and culture intersects with a number of pertinent and evolving theoretical frameworks; decolonising Indigenous methodologies (Smith 1999; Haebich 2014) ICT Indigenous pedagogies (Jorgensen 2013; Watson 2013) and Indigenous digital archiving (Nakata et al. 2008; Gardiner et al. 2011) to name a few. And there are some works which analyse the role of technology and broadly defined ‘literacy’ in remote Australian Indigenous settings similar to Gäwa in some ways (Kral 2012). Specifically, in the area of digital design and ‘architecture’, research coming out of public displays such as museums is often relevant. In this context, Srinivasan and Huang (2005) describe the concept of ‘fluid ontologies’ accommodating a ‘tighter coupling between communities’ *interests* and the browsing *structure*’ (Srinivasan and Huang 2005, p. 193), with a number of key factors facilitating this process; the knowledge content creator being directly involved with the digital structuring, making explicit the browsing and rearrangement processes available, remaining adaptive to new structures and by analysing actual histories of interactions with digital resources. In a similar vein, highlighting the agency of the content contributors, van der Velden (2010) calls for a ‘knower-centred approach’ (compared to a knowledge-centred approach) to Indigenous Knowledge Management (IKM), posing the fundamental query of how to ‘keep the relational, performative, and dynamic character of indigenous knowledge ‘alive’ in the design of knowledge management software?’ (van der Velden 2010, p. 8). Her suggestion concerns the ‘contact zone’ where Indigenous knowledge and the techno-scientific knowledge of the management software meets not being defined as one of interaction, but of ‘intra-action’ where there is ongoing entanglement; ‘with each iteration of the design, new agencies in terms of possibilities and constraints emerge’ (van der Velden 2010, p. 12). Duarte and Belarde-Lewis (2015) also offer a helpful overview of some ‘best-practice’ projects, ranging from North America to New Zealand. Particularly encouraging for the Warramiri website was to view it as an example of their identified third stage of ‘imagining’, where ‘Indigenous and non-Indigenous groups build partnerships to spread awareness and acquire formal acknowledgement of the epistemic value of local Indigenous knowledge’ (Duarte and Belarde-Lewis 2015, p. 692). Specifically, there was also endorsement that maintaining a small, localised focus was appropriate; ‘at the very basis of Indigenous thought is the understanding that Indigenous knowledge are place-based knowledge, best understood in the richness of context, through the use of Indigenous languages, and conceptualised holistically’ (Duarte and Belarde-Lewis 2015, p. 693). Indeed, such a statement could have been written as a direct summary of the Warramiri website’s foundation. In the Australian context, Dyson and Legget (2006) suggest a ‘metadesign’ methodology as the digital articles being designed are also interfaces for future design; the ‘design approach is a seeding process rather than planning exercise’ (Dyson and Leggett 2006, p. 85). Such an approach seemed to align strongly with the Warramiri website developing into a ‘live’ resource, particularly to be integrated into the ongoing curriculum and further digital development at Gäwa Christian School. Practically, Dyson and Legget also suggest a number of concrete requirements for multimedia design in Indigenous communities:

- Appropriate to Indigenous culture, particularly its oral and graphical strengths;
- Robust enough to withstand the harsh environments where many remote communities live;
- Acknowledging Indigenous knowledge protocols, security concerns over who has access to secret or sacred knowledge and intellectual property issues;
- Easy to use and navigate (given low computer literacy levels in many communities);
- Cost-effective (given the poverty of many communities);
- Allowing for diversity of communities and cultural evolution over time;
- Able to be placed outdoors at the locus of creative practice;
- Providing community control over contents and over design, development, implementation and maintenance (Dyson and Leggett 2006, p. 83).

All these theorists played an important role in ‘imagining’ the site, however, we need to insist that the relevant theory underpinning Yolŋu use of digital resources must arise from specific Yolŋu theorists and/or those who have spent considerable time understanding and applying Yolŋu knowledge systems. This is due to the profound variations of language and epistemologies even within Australian Indigenous communities and certainly within the global Indigenous community. Thus, the work of Christie, long-term teacher, linguist, lecturer and preeminent philosopher in regards to Yolŋu language and culture, provided the extensive theoretical backbone of our website project. Somewhat akin to Yolŋu knowledge-understanding progressing from ‘outside’ to more sacred and complex ‘inside’ truths, his work has progressed from the Bothways philosophy of education (Marika-Munuŋgiritj and Christie 1995; Christie 1997, 2007a) to elucidating a research methodology that genuinely incorporates Yolŋu philosophy; a ‘transdisciplinary’ approach of working at the interface of western-academia and Indigenous Knowledge systems, attempting ‘both this and that at the same time’ in terms of generating research ‘results’ but retaining the Yolŋu emphasis on solutions to real issues; research that ‘within the Aboriginal world is respectful, respectable and useful’ (Christie 2006a, p. 80). It is also ‘generative’ as the research serves a local purpose, it ‘works to generate change practices through research position’ (Christie 2013, p. 4). In particular, his long-term commitment over the last two decades to continue to work with Yolŋu practitioners has also enabled him access to emerging Yolŋu philosophies in regards to digital technology.^1^ In fact, Kathy Guthadjaka helped inform aspects of his work in prior projects and articles.^2^ Importantly, Christie and connected researchers produced a range of articles from 2004 to 2014, based on a number of large-scale digital projects which highlighted the tension between the benefits of incorporating the use of digital technology in Yolŋu intergenerational transmission knowledge practices and the inherent dangers and contradictions of databases and web-based mediums. It is these works we now synthesise and structure as our foundational theoretical paradigm.

### Benefits of digital resources

One starting point is to note that many Yolŋu have been utilising sound and visual recordings for their own political/religious purposes for a long time, with stories of cassette tapes being carried around in sacred dilly-bags and buried for later use emanating from the 1960s, less than 40 years after first contact with *balanda* (Yolŋu for ‘white person’) pastoralists and missionaries (Christie 2005a) There is also a clear desire to repatriate photos and audio files from older anthropological collections to their proper authorities. In particular, the potential for technology to aid the intergenerational transmission of language and culture is quite apt; ‘continuing the work elders have always done in Aboriginal family groups—using whatever resources come to hand in the work of regenerating clan and place as one, so as to ensure the continued health and wellbeing of both the land and the people’ (Verran and Christie 2007, p. 215). There are a number of advantages with digital technologies over more traditional, print-based resources. The focus on the aural/visual means print literacy is not as mandatory, making the resources more inclusive. The potential for many people to be involved in the performance/production and the hyperlink connectivity of digital resources also encourages a multiperspective/multidimensional domain typical of Yolŋu knowledge production instances (Christie 2001). Also, due to the increasing prevalence of Indigenous townships, and the associated lack of opportunity to visit and live ‘on country’, digital resources concerning ancestral estates and linked areas—of interactive maps and similar programmes—enable the land to function as its own database, to maintain ‘important distinctions which need to be made between groups of people in ceremonial practice, song or art’ (Christie 2007b, p. 34). And, lastly, there is the sheer ‘engagement’ factor that young people have with digital and emerging technologies, over and above, at times, their desire to learn from their elders directly (Christie 2004).

At Gäwa, there were a few other issues which also led us to think that developing the Warramiri website was appropriate. Firstly, as mentioned, many of the resources were old and were in forms such as audio cassette tapes that were deteriorating and/or were no longer readily utilised. There were scores of hours of audio-recordings of Warramiri elders (mostly passed away), so if those tapes were irreparably damaged the information would be lost forever, and in 10 years time, would there even be cassette players available? Secondly, Kathy Guthadjaka had an array of resources on her trusty Macintosh, but they were filed according to memory only, and though new and amazing resources were consistently conjured up for class use that perfectly suited the unit being studied, in terms of developing a *curriculum* that could be planned for in advance, and Warramiri literacy built from some kind of ordering was seen to be necessary. (As we shall unpack, this assumption that some kind of ‘order’ was needed is problematic and one of the key tensions in the theory and within our Warramiri website project). Lastly, a digital resource was felt to be particularly needed at Gäwa for there were many times when neither Kathy Guthadjaka nor any other Warramiri speaker was available for ‘team teaching’ in the senior classroom, so a digital bank of resources which the mainstream, *balanda* teacher could continue with, at least in some way, was seen as a preferable option than having no Warramiri language and culture input during these times.

### Dangers of digital resources

However, Christie also notes a large array of pitfalls in the use of digital technologies. A fundamental notion is that collections of resources require a certain structuring, ordering and prioritising that is inconsistent with Yolŋu epistemology and ontology. Established as an overriding position, the rub of the problem is that:

In the Yolngu world it is not so much the case that every reality has an inherent structure, but rather that every structure can be seen to inhere in a whole range of realities…the world in this sense could be seen to be endlessly structurable and humans use language to re-construct what is always a human meaning, using the lines and webs of connectedness and the names created and handed down by the ancestors to render our experiences meaningful in human terms (Christie 1994, p. 27).

This quote opens up two major issues for consideration. Firstly, by imposing a structure on the website, or database, immediately other structures are precluded, which, in their right time and place are just as valid for Yolŋu knowldege transmission. Furthermore, the process of ordering or using of metadata to identify and recall information under search parameters actually becomes a process of codifying that particular view of reality. As a form of reading-back-reality, or ‘reverse bootstrapping’ (Christie 2005a), we can thus fall into the trap of producing ‘a scientific model of the world which has its shape not because the world is so, but because this is the nature of our data structures’ (Christie 2004, p. 8). Overall then, databases, or structured websites give off the impression that they are theory-neutral, masking the technical and political decisions made in the ordering and structuring process which, in fact, work against the very Indigenous ontology of multiple connection realities they are established to support (Christie 2004). And at their worst, they can undermine the localised, primary purpose of the knowledge (‘who is telling the story, why they are telling it here and now and how the story fits into the wider networks of kinship, art, music, ceremonials and philosophy’ Christie 2005a, p. 62) by purporting to reveal how the information might be relevant to *any* person at *any* time.

Secondly, for Yolŋu, the actual process of doing knowledge is a productive one, a ‘human’ process where language does not *represent* the world, but ‘talking, singing, crying, dancing and painting all actively participate in the creation of new worlds as they have always done since the ancestors first talked, sang and danced the world into existence’ (Christie 2005a, p. 64). Christie summarises this as a ‘performative epistemology’, whereby knowledge (as opposed to identity which comes from your own family, kin, language, and land) is *produced* in conjunction and after negotiation with people of connected, but ‘different’ backgrounds (Christie 2001). That is, you preserve your own identity as you do knowledge together, by ‘identifying, acknowledging and actively maintaining the differences of language, dance, art, etc., among various contributing totemic groups’ (Christie 2004, p. 5). Of course, ceremonial performances are the prime example of this performative epistemology as no two ceremonies can ever be exactly the same; Yolŋu clan leaders negotiate the content, ordering and timeliness of performances as ‘refigurings of ancestral events to reflect current contexts’ (Christie 2005a, p. 64). Therefore, under such an epistemology, a digital resource can never properly be viewed as containing ‘knowledge’ to be transmitted to Yolŋu students or interested others. Indeed, it is merely information: a ‘series of ones and zeroes… a representation or artefact of an earlier act of knowledge performance/production’ (Christie 2004, p. 4). Thus, the real danger is in thinking that in creating a database or website, a repository of Indigenous knowledge has been formed, whereas, in truth, what exists is only a ‘memory resource containing assemblages of traces of previous truth-claim episodes’ (Christie 2005a, p. 64).

Lastly, also emerging from the nexus between identity and knowledge is the reluctance among Yolŋu to assume ownership and thus, authority over information in digital resources purporting to represent the broader community. It is not so much an unwillingness to share knowledge with the rest of the world (although these intellectual property issues are concerning; Guyula and Gurruwiwi 2010), but due to the key, negotiated aspect of knowledge production, many are ‘keen to avoid being held responsible in any way for the management of, and particularly the access to the resources of others’ (Christie 2007b, p. 33). As all Yolŋu townships are a complicated mix of languages and clans, with traditional owners, ‘caretaker’ clans and inter-clan kinship networks all relevant, it makes any digital resource, even at a local ‘community’ level, a complex matter to resolve, and brings into question whether the finished ‘product’ will ever really be utilised in a meaningful manner. One of the Yolŋu who informed much of the theorising vividly likened the attempt at a generic ‘Knowledge Centre’ database at Galiwin’ku to that of the old mission cemetery ‘where lifeless but potent bodies of all different connections were amassed together unsupervised’ (Christie and Verran 2013, p. 307).

### Indigenous digital resources: theory

How then, can well-intentioned *balanda* partner in the creation of databases and websites to assist in the intergenerational transmission of language and cultural knowledge without compromising the ontological and epistemological frameworks inherent in such negotiated-performative practice? Christie posits:

An alternative Australian Indigenous epistemology may emphasise the performative nature of knowledge, its negotiation from multiple perspectives and multiple modes of presentation and prosecution, and its fundamentally narrative base (Christie 2006b, p. 291).

Thus, there are three elements to consider. Firstly, to emphasise the ‘performative nature of knowledge’, a digital resource will have to acknowledge explicitly that the information loaded on to the database or website is not a ‘definitive’ version (especially so if it is a recording of a previous performative knowledge production occasion itself, like ceremonial activity). It may also mean creating the resource to serve a particular educational purpose or function, not something to be available online ‘forever’. In other words, designers should start with the ‘*uses* of digital artefacts as the framework for system development. Who will use it, how and where?’ (Christie 2004, p. 10).

Secondly, in terms of the ‘multiple perspectives and multiple modes of presentation and prosecution’ as above, Christie relies on the concept of *garma*. This term is now well known as the name of the annual Yolŋu conference/festival which attracts considerable artistic and political interest both throughout Australia and internationally. But originally, the term *garma* was connected to the Yirrkala ‘Bothways’ curriculum of the 1980s, where it was used to define the intercultural interplay between ‘western’ and Yolŋu education systems being developed at the school (Marika-Munuŋgiritj et al. 1990). *Garma* was appropriate because it was a pre-existing Yolŋu concept of inter-clan negotiation, an ‘open ceremonial ground where different groups come together for negotiated performances where ancestral histories are performed in the context of contemporary issues, and where current truth claims are presented and assessed’ (Verran and Christie 2007, p. 218). *Garma* was developed by Yolŋu educators themselves in a number of powerful and fascinating elucidations outlining the key applicability of the concept to education (Marika-Munuŋgiritj et al. 1990; Marika 1999). In relation to digital futures, Christie furthers the process from a literal *garma* of land, through the metaphoric *garma* of curriculum, to a new literal/metaphoric virtual *garma* or website, where digital resources ‘need not be in real time, but must be ordered… the site becomes a garma through the careful implementation of protocols for performance, and the provision of rich networks of (hypertextual) linkages among these performances’ (Christie 2001, p. 48). Thus, many perspectives are encouraged and negotiated, but with Yolŋu ownership of permissions, where ‘people are invited to enter, and observe, and do their thing under supervision’ (Christie 2001, p. 49).

Lastly, in relation to retaining a ‘narrative base’, or, in fact, an ongoing narrative function, it is more than simply including examples of ‘stories’ amidst the other information on plants, animals, land features, seasons, etc. The entire resource must help to enable the drive towards performative epistemology which has narrative at its core. In this regard, Christie advocates ‘flattening-out’ of the metadata which stifles connections and narrative possibilities as the crucial design methodology, and highlights the understanding that digital literacy or narrative is a two-edged sword with the productive element feeding back onto and enhancing the receptive one. In other words, Yolŋu ‘will learn to read databases profitably for their own purposes as they learn to write them’ (Christie 2004, p. 11).

### Indigenous digital resources: practice

Practically, how do we ensure that a database/site, with its ‘architecture and structure, its search processes and interfaces, its ownership’ (Christie 2004, p. 8) remains married to the Yolŋu ontology and epistemology? Christie’s solution is to strive for ‘postcolonial databasing’ (Verran and Christie 2014) of ‘fluid ontologies’ (Christie 2006b) and ‘located accountability’ (Verran et al. 2007). In a humble contrast, Christie readily acknowledges that his first attempts at collating and storing stories both on CD format and online in the 1990s for the ‘Yolŋu Studies’ programme at Charles Darwin University was ‘an essentially non-Aboriginal way of structuring and presenting Aboriginal knowledge’ (Christie 2005b, p. 55), and thus, he positions two alternatives for future development. Either, developers ‘encode the complex connectivity of Yolngu knowledge in the data structure… (or) do away with the attempt to hard wire the relationality into the database… collapse the metadata categories and create the conditions whereby Indigenous owner-users can learn to invoke and encode for themselves’ (Christie 2005a, p. 61). Whilst acknowledging the first option may be possible, he warns that ‘designers, in Australia have enjoyed very little success in their attempts to replace an embedded western classificatory system, with an imagined Indigenous alternative’ (Christie 2006b, p. 290) and that even approaches which highlight the social/kinship perspective rely on western categorisation prioritisations (Verran et al. 2007). Thus, he favours the ontological ‘flattening-out’ approach to the metadata; that is, the second alternative of purposively allowing digital artefacts to sit ‘nameless’ on sites and databases, with connections and narrative meanings to be drawn by the users themselves. The development of the TAMI (Text, Audio, Movies and Images) prototype software was a systematic attempt to embody the principles outlined above. The key features of TAMI were:

- Single interface screen enabling searching, building of presentations, uploading and metadata functions.
- Workspace enabling simultaneous viewings of resources.
- Only initial metadata distinction as between text, audio, movies and images. That is, no other ‘fields’ to discriminate.
- Folders created and different metadata attached as users decide.
- Search capacity through full ‘thumbnail’ visuals, not just text based (Verran et al. 2007).

**Fig. 2:**
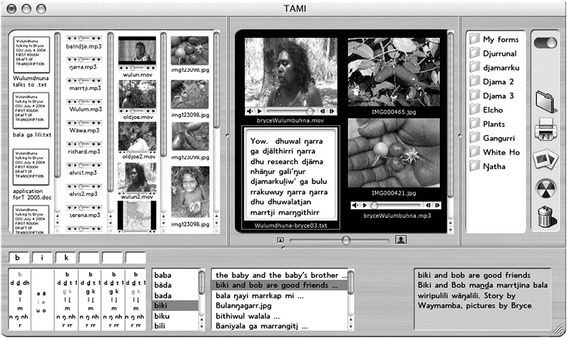
TAMI template

Thus, the TAMI database was a culmination of the theoretical position slowly developed since at least 2001, purposively to allow ‘bringing together unstable configurations for a particular collaborative performance, but without destroying their ability to fall apart, to return to their full potential for reconfiguration’ (Christie and Verran 2013, p. 307). Ultimately, Christie championed small, localised, highly personalised collections with minimal location protocols whose ‘owners know what they contain and exercise the same stewardship as they do over their ‘natural’ resources’ (Christie 2008, p. 285) (Fig. 2).

## Results/Discussion

### The Warramiri Website (2011–2015)

Although we were unaware of the TAMI work at the time, Kathy Guthadjaka’s computer was an example of a TAMI system, certainly. Ironically, what Christie advocates, in the end, was a digital template that already existed at Gäwa. It was contained and ‘owned’ by her alone, and she continually made decisions as to which specific resources could/should be brought into the public (garma) space that was the ‘senior’ class, only for them to fall back into her own filing system of multisaved entries (as opposed to categories) to be considered for use in another future knowledge-production instance. Perhaps, if we had read all of the work Christie and others had compiled *before* we began the website development, it may never have eventuated! However, despite the synthesis presented above, we grappled with the theoretical framework having already committed to the construction of the Warramiri website; theory and practice developed together over the years 2011–2015. Nevertheless, we argue now that the situation at Gäwa bears some marked differences to the environments postulated in aspects of Christie’s work. Thus, this last section tells the story of the website through the three categories of Christie’s alternative Indigenous epistemology of digital databases/websites previously outlined, highlighting the areas of congruence and problematising the areas of departure.

### Performative knowledge nature

Clearly, the concept that Yolŋu knowledge is made together after negotiation of kinship and inter-clan relationships are carefully worked through is as true for the Warramiri at Gäwa as it is anywhere else in north-east Arnhem Land. Indeed, not all the community members and students at Gäwa are Warramiri at all. However, they are all *connected* to Warramiri in a specific way, often in the *yothu-yindi* or *märi-gutharra* inter-clan kinship relationship (McIntosh 1997). And these relationships entail detailed obligations in terms of caring for and maintaining Warramiri land and overall affairs. For example, a number of significant funeral ceremonies have been held at Gäwa over the preceding few years and this complex, at times strained, process of negotiation/performance was clear to see, even for largely ignorant *balanda*. Likewise, Elders and students often return to Galiwin’ku and sometimes travel to further townships to attend ceremonies and memorials of other connected clans. Nevertheless, the clear focus of day-to-day learning for the students at Gäwa concerns their specific Warramiri clan upbringing. Or in Christie’s terms, there is a focus on *identity* as opposed to knowledge production. As he notes, in the overall epistemology ‘there is a difference between identity and knowledge. Identity can be learnt within the context of your own family, your kin, your traditions, your language and your land. Knowledge production takes place as a result of negotiation between people of different backgrounds’ (Christie 2001, p. 37). Gäwa was primarily established so that young Warramiri could grow up Warramiri. Indeed, the aim was to learn of their own identity, on their own land, in contrast to Galiwin’ku with its overlapping of many clan groups now residing there and the more generic ‘Yolŋu’ culture and language taught at the large community school. One of the specific concerns is that Warramiri children (and all other Yolŋu children on Elcho Island) currently grow up speaking Djambarrpuyŋu, as the form of *lingua franca* of the Yolŋu languages. But this may not, in fact, be either their mother’s nor their father’s clan language. Thus, for the intergenerational transmission work being done at Gäwa, priority is placed on learning from the Warramiri land and language that is the foundation of Warramiri identity.

However, there is still a ‘performative’ aspect, but it is more focused on the performance of Yolŋu as they relate to their ancestral estates; as they holistically learn ‘on country’. Kathy Guthadjaka has explained this emphasis previously, when at Gäwa the young people ‘know the land and the breezes, and the water, what time the tide will be in, when it will be out, because they are learning on country, and he grows with them, by means of that learning’ (Guthadjaka 2010, p. 27). Thus, there is certainly still a focus on the interactive nature of identity, but the interplay between the *land* of Gäwa and the Warramiri is where the performance plays out, on a daily basis. However, this insight did not initially help us around the fact that there exists a ‘disjunction between the structured information to be found on a computer, and the integrated, holistic, lived and performed knowledge of Aboriginal people on country’ (Christie 2004, p. 10). Indeed, intuitively we found that other sites (though carefully researched and beautifully presented) such as ones which represented Gupapuyŋu kinship, or areas around Ramingining, inevitably structured knowledge around ‘topics’ (kinship, story, song, art, etc.) originally formed from anthropological frameworks.^3^

**Fig. 3:**
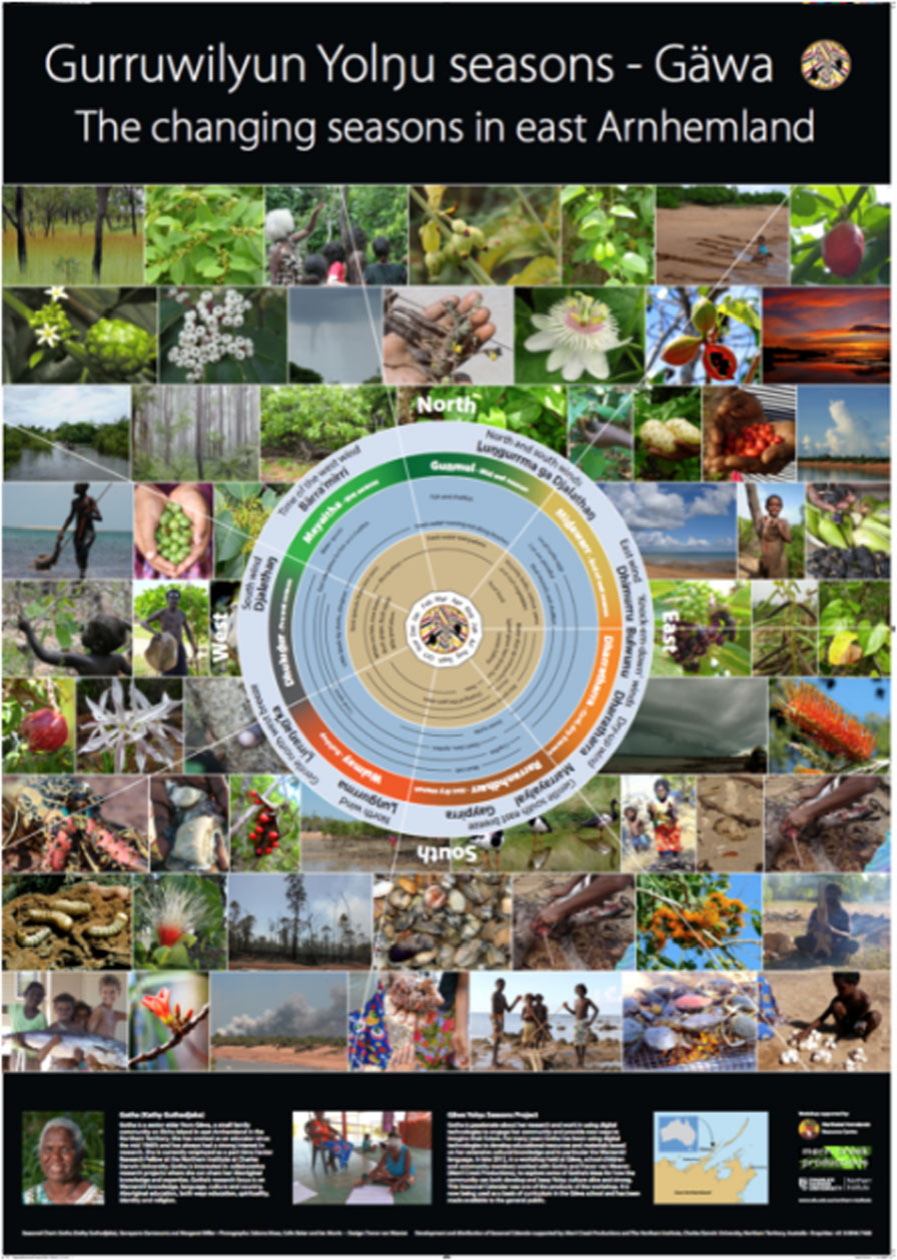
Gäwa season calendar

But we were determined to attempt the first option mooted by Christie, a structuring that *did* reflect both the daily life of the young Warramiri and the identity-forming priorities of their Elders. The first significant breakthrough occurred in 2011 when we reflected on the fact that the seasonal-cyclical nature of learning ‘on country’ was the one constant, and indeed, the beginning discussion for virtually every Warramiri class we had taught together. Interestingly, Warramiri include the season of *Wolmay* in their ‘calendar’, a concept not readily acknowledged in more generalised Yolŋu seasonal information (Davis 1989). Our thinking developed from here; could we perhaps combine the appeal of a seasonal ‘calendar’ chart with the website itself? Could we then structure other resources loosely into which ‘season’ they best aligned? For the first few years, we proceeded with this template, the ‘Home’ page was a cyclical calendar (based on the beautiful poster chart designed in conjunction with Trevor van Weeren), whereupon clicking on a segment-season, you would be taken to further resources (stories, songs, plants, animals, hunting information) related to that season. This enabled a structure that seemed to match the holistic nature of daily life at Gäwa, but that was still quite flattened-out, allowing configurations of resources and performances for local, specific purposes (Fig. 3).

### Multiple perspectives and multiple modes

As the seasonal approach to the website gathered momentum and we worked on ‘positioning’ various resources into the framework, it became clear that if it were truly to function as the foundation for the school’s curriculum, we also needed a variety of texts, at various literate and visual levels, to accommodate the students ranging from ﻿age 5 to 15 who attended school. In a sense then, we put something of a twist on Christie’s criteria of multiple perspectives. Where he had imagined a virtual garma where connected but specific clan (and other) identities were showcased after negotiation, as the Warramiri site was more concerned with *identity* rather than knowledge production, our multiple perspectives/modes concerned multiple ways of approaching the same theme or topic. In particular, as Elders discussed in the more formal interviews connected with the website research how children learnt before the introduction of formal schooling, in terms of consistently observing their family’s participation in routines on country and ceremonial performances, year after year, we embarked on developing a related ‘overlearning’ cycle to the website. That is, under each season, various resources would be available, some aimed at the very young, some at primary aged children and some for senior students. Thus, the yearly ‘Language and Culture’ curriculum at Gäwa could always ‘turn’ (*gurruwilyun*) around the seasonal cycle, but students would study deeper (‘inside’) and more literate Warramiri resources as they grew older and were deemed ready by the Elders. In this way, the site could function as a kind of simulacrum of the traditional Indigenous cyclical enculturation process into maturity.

**Fig. 4:**
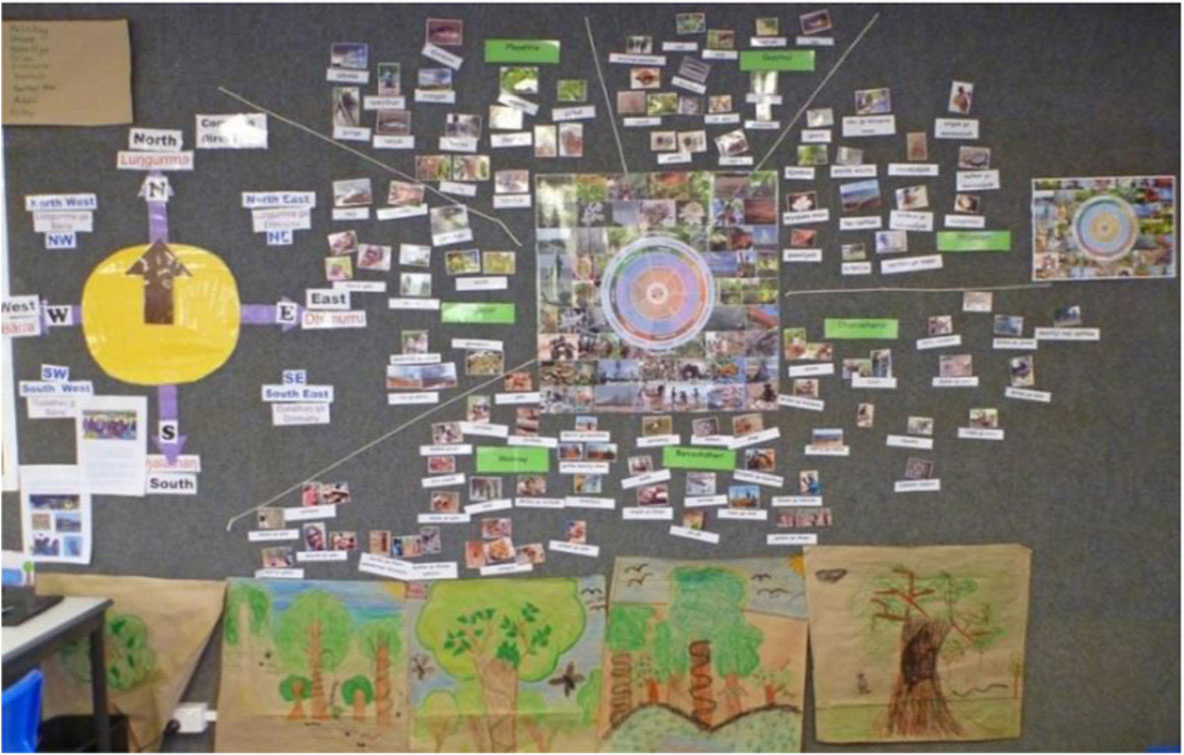
Gäwa season calendar in the school curriculum

The other strength of this approach was that it gave impetus to the *older* notion of garma; the symbol of the Bothways curriculum of intercultural interplay between Yolŋu and mainstream educational priorities. In 2013, a fine example of this occurred when, using the seasonal template, a combined unit was designed around the Macassan visits to Warramiri land arriving on the ‘Northwind’ with associated stories and information around specific sites, plants, words and ceremonies introduced. But the unit also explicitly linked with the new History: Year 4 component of the Australian Curriculum, where students learn of ‘the diversity of Aboriginal and Torres Strait Islander Peoples, their connection to place… and their contact with other societies’, specifically being referred to ‘the trade between the Macassans and the Yolngu people’ in the Knowledge and Understanding content descriptor ACHASSK086 (Australian Curriculum and Reporting Authority 2016). And when it comes around to *Mayaltha* season again (roughly January to March) at the start of the following school year, a connected but more in-depth literate unit involving the ancestral dog *Djuranydjura* could be studied which also involved Macassan stories, songs and excursions to local ‘sacred’ sites with the potential for ‘inside’ sessions led by elders (van Gelderen, B., and Guthadjaka, K.: Creating the Warramiri website: some bala-räli conversations, submitted). Or perhaps the focus could turn to some of the digital audio files which sit under the *Mayaltha* season that focus on the new growth of plants and fruits and dangers (literal and metaphoric) of the stone fish and stingray in the muddy waters. The ultimate point is that there are a number of resources and layers within each season and decisions on which area to emphasise can be negotiated with Elders and school teachers together. Crucially, through such an ‘overlearning’ cycle, the Warramiri children will progress in ‘language and culture’ as the seasons and years turn (Fig. 4).

A further, very different area of ‘multiple perspectives’ worth noting concerned the position that certain perspectives were viewed as unhelpful or even hurtful; to be excluded. In the age of digital ‘access’, the Warramiri Elders throughout the formal research interviews were often more concerned with digital *restriction*. Firstly, there were restrictions *from* influences that were eroding the ongoing work of transmission of culture and language. After undertaking community research on this topic, Kathy Guthadjaka presented findings to the annual ‘Over the Top’ conference for the Northern Territory Christian Schools in 2012:

In Arnhem Land the spring tides have brought lots of foreign things to our shores. Junk and rubbish mostly. The same tides have taken away our children and we have *milkarri*; crying songs about the loss of children to Darwin and other faraway places. The *milkarri* you will hear now is just one of these songs (Guthadjaka 2012).

Specific examples then included the ‘rubbish’ of American-influenced rap music and bad language, the gambling and aimlessness in town and the fighting and swearing when kids return from town after ‘running wild’. It was an endorsement for homeland living overall, but the role of outside, often digital influences through the internet was highlighted as containing a dangerous potential to function as the outgoing tide of pulling children away from their heritage.

But there was also a restriction *to* digital information that evolved over the course of the project. At first, the intention of the Warramiri website was for both the Gäwa community and interested outsiders (particularly other Australian schools) to use the site to learn something of Yolŋu history and culture. However, as the site progressed and a number of sad events also impacted on the Gäwa community, overall trust deteriorated that outsiders would not appropriate the images and video/sound files on the site for their own purposes. In the end, in 2016, a password was added to the site as protection. Indeed, like Christie, we found that ‘processes of exclusion are as important as those of sharing in the work of keeping land and culture strong’ (Christie 2006b, p. 286).

### Narrative base

The last of Christie’s elements for an alternative Indigenous epistemology regarding digital learning focussed on how the narrative foundation of knowledge production must remain at the fore. His solution was to do away with virtually all the metadata or structure to allow users to create their own connections; to create local, ever-changing, specific narratives. As has been discussed, we instead chose to utilise the seasonal cycle as a structure, attempting to align with the holistic life at Gäwa. However, it soon became apparent that many of the resources did not neatly fit in to any of the season segments. There were many interconnected stories and songs, tracing ancestral movements across land, connecting to significant totemic characters and items. As part of the broader Warramiri website project, we also undertook whole-of-community consultation around ‘two-way’ education at Gäwa, in an attempt to clarify appropriate local pedagogies when it came to Warramiri/Djambarrpuyŋu/English usage as well as learning styles, team teaching, intercultural curriculum, etc. (van Gelderen 2016). One of the clear messages to emerge was the primacy of learning ‘on country’. When asked about whether teaching Warramiri should happen at home or at school, one of the Elders and Ceremonial Custodian for Gäwa replied:

Yes, in the school and when they finish (the school day), then they learn the Yolŋu way. It must be there, on their own land. Yes, there, on their own land (Gäwa), not on somebody else’s land. The school is at home, it’s all home. They learn Yolŋu way, the culture at ‘home’. It is all the same, it is home. Home, home, home…

Profoundly expressed here, similar place-based, on country sentiments came through in virtually every interview, and community members also spoke about other traditional Warramiri sites on the mainland at Dholtji and Maṯamaṯa and islands through the Wessels. Ben van Gelderen’s notes from later that night were: *In my desire for the seasonal structure to incorporate as much of the traditional knowledge as possible, I have overlooked how each parcel of land contains the ‘truth’ necessary for the education of Yolŋu. The Land incorporated and gave birth to both the people and the language (and a number of stories mentioned today included details of how people changed from speaking one Yolŋu language to another at certain places) and thus, naming and knowing land is of utmost importance* (van Gelderen 2016). Thus, after further consideration, we felt it was only appropriate that the priorities of the Elders from the more formal research recordings were specifically reflected in the Warramiri website structure itself. The role of land—and the inherent connection to language that entails—is so paramount for the students in understanding their Warramiri identity that, in 2013, we altered the home page from the seasonal calendar to a more traditional ‘Welcome’ page with a list of other ‘Title’ pages in a loose hierarchy across the heading bar: Land-People-Language, then followed by Seasons-Men/Women-Philosophy. It was a significant change, and it was a little sad to depart from the seasonal-cycle structure which had been at the foreground of our approach for so long and visually gave off such a clear ‘alternative’ appeal.

However, the season focus was still present and practically within the site, a majority of the visual resources were still housed within these pages. In this sense, the site was akin to the beautifully presented ‘Yan-nhangu Atlas’ which was the culmination of a long-standing project in the homelands of the Crocodile Islands, similar in many ways to our own project; interested *balanda* researcher collaborating with a Yolŋu elder over a number of years, to preserve and document her particular clan languages, history and seasonal life-cycles, all as a legacy for the future generations.^4^ This ‘illustrated dictionary’ also has much knowledge structured around the seasons, but includes distinct ‘chapters’ on language, history and specific land sites as well (Baymarrwana 2014). The Warramiri website had an almost identical purpose, and structurally it was now also quite aligned, but with the focus on ever-adapting, digital resources. Furthermore, from 2013, each of these new Title pages then began a story of their own, with much reflection on the applicability of resources and language choices to frame each area (in both Warramiri and English). And the fundamental point to note for present purposes is that each page still opens up to reveal numerous digital resources, but without further sub-categories, and a number of the resources are stored in multiple pages as deemed relevant. The practical result meant that the site now ‘looked’ like many of the other sites around Yolŋu language and culture; perhaps we had succumbed to the unwise move of inscribing ‘Aboriginal taxonomies or patterns of relatedness (person–clan–language–place–totem for example) into information architecture’ (Christie 2006b, p. 290). But it was as a direct result of the emphasis that local Warramiri Elders placed on these areas, and importantly, it was also designed in an attempt to reclaim the narrative foundation of the website. The seasonal cycle was clear and powerful, but the narratives around land, people and language (the key stories the Elders are so keen to preserve and transmit) were positioned as utterly foundational.

## Conclusion

**Fig. 5:**
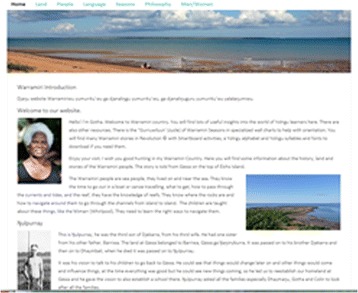
The Warramiri website homepage

The Warramiri website had a long gestation period, but this is something we are proud of. We intentionally wished to ‘work slowly and allow new ideas and practices to emerge here and how, and grow slowly through mutual respect and a history of shared experience’ (Christie 2009, p. 32). We wrestled with the nature of structures, multiple perspectives and performative knowledge practices, and even though the website on the surface appears relatively simple to negotiate, we believe some of the complexity of Warramiri ontology and epistemology underpins it. But even more crucial is the fact that the entire website was envisaged and developed by the people who desire to use it, and by utilising a generic and reasonably easy template to manipulate, it continues to change. The Warramiri website is not complete, it is intentionally incomplete and it will continue to alter and grow according to community wishes. The final step (which began in April 2017 with professional development days for Gäwa staff on the site and its underlying philosophy) is to truly integrate the various resources with the school curriculum through a Bothways negotiation between community Elders and current teachers. This, we believe, is the ultimate narrative foundation, in that stories are told by people, and the people telling the stories at Gäwa are the Warramiri who have total command over the digital resource that is the Warramiri website. As Christie concludes, the ultimate goal of the alternative epistemology is that ‘the distinction between the programmer and the user begins to dissolve, and the accountability of each is located in the relations which make the technologies useful’ (Christie 2005a, p. 65). At Gäwa, we believe this process has only just begun (Fig. 5).

## Footnotes

1 Significant multiyear research projects such as ‘Indigenous Knowledge Traditions, and Digital technology’ (http://www.cdu.edu.au/centres/ik/ikhome.html), ‘Teaching from Country’ (http://learnline.cdu.edu.au/inc/tfc/index.html) and ‘ICT and Capacity Building in Remote Communities’ (http://www.cdu.edu.au/centres/inc/) all directly involved Yolŋu co-researchers and digital learning.

2 She was co-author in Guyula et al. 2010 and Christie formally acknowledges her contribution to his other works in Christie and Greatorex, 2009 (Gambling report), Christie and Campbell, 2013 (Housing report) and Christie, 2010, (Teaching from Country), 2011, (Gifted and Talented education), 2014 (Community development).

3 See Gupapuyŋu http://www.gupapuyngu.com/wegupa.html, Twelve Canoes http://www.12canoes.com.au/

4 Indeed, we were unaware of this project until the actual ‘Atlas’ was delivered to the school at Gäwa. Despite being a beautifully illustrated and researched volume of over 500 pages, it is not for sale, but is to be distributed to various Yolŋu homeland communities.
